# Supplementary material for: Optimizing the order of actions in a model of contact tracing
Source: PNAS Nexus. 2023 Jan 20;2(3):pgad003. doi: 10.1093/pnasnexus/pgad003 (PMC10013731; doi:10.1093/pnasnexus/pgad003)
Supplement: pgad003_Supplementary_Data [file pgad003_supplementary_data.pdf]

6. SI Appendix: Univariate Model

While in the basic model querying nodes in order of recency is optimal, this is not always the case in the univariate model. In the univariate model the probability of infection decays absolutely with time according to an exponential functional form.

Fixing $p_T \in (0,1]$ and $\alpha \geq 0$, for any $h \in \{0,1,\ldots,T\}$ the probability of infection is $p(h) = p_T e^{-\alpha(T-h)}$. All other parameters of the univariate model are identical to the basic model. Observe that if $\alpha = 0$ the probability of infection is constant and the univariate model instantiates the basic model. Additionally, p_T represents the probability that a node of recency T is infected.

Examining trade-offs and thresholds. As in the basic model, there is a trade-off between querying a less recent node, which provides an opportunity to significantly expand the frontier, and querying a more recent node which, if infected, returns a larger immediate benefit. However, since the probability of infection decays with time, a more recent node has a lower probability of infection. As a result, querying a more recent node does not necessarily return a higher *expected* immediate benefit. Thus choosing a node to query involves a trade-off between a node's recency and probability of infection.

The calculation of this trade-off changes with α. If $\alpha = 0$ the probability of infection is constant, so as in the basic model, querying nodes in order of recency is optimal. As α increases it eventually hits a threshold beyond which this policy is no longer optimal, at which point it is natural to wonder whether any structure remains. In fact, we show that the there is still structure to the optimal policy: the policy always queries either the most recent or least recent node available. We say that such a policy is defined by an *interleaved* priority ordering.

Definition 6.1 (Interleaving property)**.** An ordering σ on $\{0,1,\ldots,T\}$ is *interleaved* if for all $0 \leq j \leq T$, σ_j is either the maximum or minimum element of the suffix $\sigma_j, \ldots, \sigma_T$.

Observe that many different priority orderings satisfy the interleaving property. For example, an ordering that prioritizes nodes by recency is interleaved, as is an ordering that prioritizes nodes by reverse recency. Once $\alpha = \beta$, each node returns the same expected benefit, and we show that in this case any priority ordering is optimal. Once $\alpha > \beta$, a less recent node both returns a higher expected immediate benefit and has more children in expectation, and we show that in this regime querying nodes in order of reverse recency is optimal. Taken together, these results imply that the interleaving property holds for all instances of the univariate model.

Theorem 6.1. *In the univariate model there is an optimal policy defined by an interleaved priority ordering.*

Proof. The main challenge is proving that the interleaving property holds for $0 < \alpha < \beta$, which we show in theorem 6.2. If $\alpha = 0$, querying nodes in order of recency is optimal, by theorem 4.1. If $\alpha \geq \beta$, then querying nodes in order of reverse recency is optimal, as shown in theorems 6.4 and 6.5. In general, the proof techniques involve analyzing periods and follow a similar structure as the proof in theorem 4.1. □

A. Defining the optimal policy. The optimal policy is constructed via the same dynamic program as defined in eqs. (5) and (6), except the probability of infection is now determined by the function $p(h)$.

$$\sigma_0 = \underset{h \in \{0,1,\ldots,T\}}{\arg\max} \; p(h) e^{-\beta h} \qquad [9]$$

In any round $k > 0$, the prefix $\sigma_0, \sigma_1, \ldots, \sigma_{k-1}$ is fixed, and σ_k is selected from the elements not already in the prefix.

$$\sigma_k = \underset{h \in \{0,\ldots,T\} \setminus \{\sigma_0,\ldots,\sigma_{k-1}\}}{\arg\max} \frac{p(h)\left(e^{-\beta h} + e^{-\beta}\, \mathbb{E}_{Z(h)\sim D^h}\left[b(Z(h), \sigma_{k-1})\right]\right)}{1 - e^{-\beta}\left(p(h)\, \mathbb{E}_{Z(h)\sim D^h}\left[e^{-\beta \tau(Z(h), \sigma_{k-1})}\right] + 1 - p(h)\right)} \qquad [10]$$

B. Analyzing the optimal policy. We analyze the above construction via similar strategies as in the proof of theorem 4.1.

Theorem 6.2. *In the univariate model, for $0 < \alpha < \beta$, there is an optimal policy defined by an interleaved priority ordering.*

Proof. Fix $T \in \mathbb{N}$, $p_T \in (0,1]$, $\beta > 0$, and $0 < \alpha < \beta$. Let σ be the optimal priority ordering constructed via the dynamic program in section A. It suffices to show that for all $0 \leq j \leq T$, $\sigma_j \in \{\max_{i \geq j} \sigma_i, \min_{i \geq j} \sigma_i\}$.

Proof by induction. By definition,

$$\begin{aligned}
\sigma_0 &= \arg \max_{h \in \{0,1,\ldots,T\}} p(h) e^{-\beta h} \\
&= \arg \max_{h \in \{0,1,\ldots,T\}} p_T e^{-\alpha T} e^{(\alpha - \beta) h} \\
&= 0
\end{aligned}$$

Here 0 attains the maximum, since $\alpha < \beta$.

Fix $0 < k < T$. Assume that for all $0 \leq j \leq k-1$, $\sigma_j \in \{\max_{i \geq j} \sigma_i, \min_{i \geq j} \sigma_i\}$. Then there exist elements $l, m \in \{0,1,\ldots,T\}$ with $0 < l \leq m$ such that $\{l,\ldots,m\} = \{0,1,\ldots,T\} \setminus \{\sigma_0, \sigma_1, \ldots, \sigma_{k-1}\}$. Fix $h \in \{l,\ldots,m\}$ and let v be a node with recency $h(v) = h$. Then the children of v are described by a multiset $Z(h) = (Z_0, Z_1, \ldots, Z_{h-1}) \sim D^h$, where Z_j indicates the number of children of recency j. Consider a $(Z(h), \sigma_{k-1})$-epoch. Since a child is more recent than its parent, any descendant u of v has recency $h(u) \leq h - 1$. As a result, a $(Z(h), \sigma_{k-1})$-epoch involves only nodes with recencies in $\{0,1,\ldots,h-1\}$. Additionally, the prefix $\sigma_0, \sigma_1, \ldots, \sigma_{k-1}$ dictates that any node u queried during the epoch has recency $h(u) \preceq \sigma_{k-1}$. Combining these two restrictions, if a node u is queried during a $(Z(h), \sigma_{k-1})$-epoch then

$$h(u) \in \{\sigma_0, \sigma_1, \ldots, \sigma_{k-1}\} \cap \{0,1,\ldots,h-1\} = \{0,1,\ldots,l-1\}$$

Let $\Pi_l(Z(h)) = (Z_0, Z_1, \ldots, Z_{l-1})$ be the projection of $Z(h)$ onto the first l coordinates, and note that $\Pi_l(Z(h)) \sim D^l$. Following the same reasoning as in theorem 4.1, the benefit and duration of a $(Z(h), \sigma_{k-1})$-epoch are identically distributed as the benefit and duration of a $(\Pi_l(Z(h)), \sigma_{k-1})$-epoch. Call the total expected benefit b_l and observe that it does not depend on h.

$$\mathop{\mathbb{E}}_{Z(h)\sim D^h}\left[b(Z(h), \sigma_{k-1})\right] = \mathop{\mathbb{E}}_{Z(h)\sim D^h}\left[b(\Pi_l(Z(h)), \sigma_{k-1})\right] = \mathop{\mathbb{E}}_{Z(l)\sim D^l}\left[b(Z(l), \sigma_{k-1})\right] = b_l \qquad [11]$$

DRAFT

Likewise, since the duration is identically distributed, the expected pre-multipliers are equal. Call this pre-multiplier γ_l, and observe that it does not depend on h.

$$\mathop{\mathbb{E}}_{Z(h)\sim D^h}\left[\gamma(Z(h),\sigma_{k-1})\right] = \mathop{\mathbb{E}}_{Z(h)\sim D^h}\left[\gamma(\Pi_l(Z(h)),\sigma_{k-1})\right] = \mathop{\mathbb{E}}_{Z(l)\sim D^l}\left[\gamma(Z(l),\sigma_{k-1})\right] = \gamma_l \qquad [12]$$

Given the prefix $\sigma_0, \sigma_1, \ldots, \sigma_{k-1}$, the element σ_k is selected by comparing (h, σ_{k-1})-periods for h not in the prefix, that is, for $h \in \{l, \ldots, m\}$. Therefore for all h in consideration eqs. (11) and (12) hold. Applying these identities and the definition of $p(h)$ to eq. (10),

$$\begin{aligned}\sigma_k &= \mathop{\arg\max}_{h\in\{l,\ldots,m\}} \frac{p(h)\left(e^{-\beta h} + e^{-\beta} b_l\right)}{1 - e^{-\beta}\left(p(h)\gamma_l + 1 - p(h)\right)} \\ &= \mathop{\arg\max}_{h\in\{l,\ldots,m\}} \frac{p_T e^{-\alpha(T-h)}\left(e^{-\beta h} + e^{-\beta} b_l\right)}{1 - e^{-\beta} + p_T e^{-\alpha(T-h)-\beta}(1-\gamma_l)}\end{aligned}$$

Define the function $I_l : \mathbb{R} \to \mathbb{R}$, where

$$I_l(x) = \frac{p_T e^{-\alpha(T-x)}\left(e^{-\beta x} + e^{-\beta} b_l\right)}{1 - e^{-\beta} + p_T e^{-\alpha(T-x)-\beta}(1-\gamma_l)} \qquad [13]$$

Then

$$\sigma_k = \mathop{\arg\max}_{h\in\{l,\ldots,m\}} I_l(h) \qquad [14]$$

By lemma 6.3 I_l is convex. This implies that the maximum value of I_l on any closed interval is attained at an endpoint. Therefore

$$\sigma_k = \mathop{\arg\max}_{x\in\{l,\ldots,m\}} I_l(x) = \mathop{\arg\max}_{x\in[l,m]} I_l(x) = \mathop{\arg\max}_{x\in\{l,m\}} I_l(x) \qquad [15]$$

As a result, $\sigma_k \in \{l, m\}$, so $\sigma_k \in \{\min_{i\geq k}\sigma_i, \max_{i\geq k}\sigma_i\}$. Therefore σ is interleaved. □

The following lemma completes the above proof.

Lemma 6.3. *I_l is convex.*

Proof. Fix $T \in \mathbb{N}$, $p_T \in (0, 1]$, $\beta > 0$, and $0 < \alpha < \beta$. Fix $b_l, \gamma_l \geq 0$. It suffices to show that I_l' is non-decreasing.

Set $C_0 = p_T e^{-\alpha T}$, $C_1 = e^{-\beta} b_l$, $C_2 = 1 - e^{-\beta}$, and $C_3 = p_T e^{-\alpha T - \beta}(1 - \gamma_l)$. Given these parameters, we can rewrite I_l as

$$I_l(x) = \frac{C_0 e^{\alpha x}\left(e^{-\beta x} + C_1\right)}{C_2 + C_3 e^{\alpha x}}$$

Then

$$I_l'(x) = \frac{C_0 e^{\alpha x}}{(C_2 + C_3 e^{\alpha x})^2} \cdot \left[C_2(\alpha - \beta)e^{-\beta x} - C_3 \beta e^{(\alpha-\beta)x} + \alpha C_1 C_2\right]$$

Observe that $C_0, C_2, C_3 > 0$ and $C_1 \geq 0$. Examining $I_l'(x)$, note that the third term is positive and has no dependence on x. Since $0 < \alpha < \beta$, the first two terms are negative and both decrease in absolute value as x increases. Thus $I_l'(x)$ is non-decreasing, and therefore I_l is convex. □

In the special case when $\alpha = \beta$, the order in which nodes are queried has no effect on the total expected benefit, and any policy is optimal.

Theorem 6.4. *If $\alpha = \beta$, any policy is optimal.*

Proof. Fix $T \in \mathbb{N}$, $p_T \in (0, 1]$, and $\alpha = \beta > 0$. It suffices to show that, starting from an arbitrary state S, any arbitrary policies P_1 and P_2 achieve the same total expected benefit.

Let $b(S, P_1)$ and $b(S, P_2)$ be random variables indicating the total discounted benefit accumulated by running P_1 and P_2, respectively, from the initial state S. Since $\alpha = \beta$, at any step t the expected discounted benefit of querying any node in the frontier is

$$p(h)e^{-\beta(t+h)} = p_T e^{-\alpha T + (\alpha-\beta)h - \beta t} = p_T e^{-\alpha T - \beta t}$$

As a result, a policy that queries a node at step t receives an expected discounted benefit of $p_T e^{-\alpha T - \beta t}$. Thus the total expected benefit a policy achieves is defined by the first step in which the frontier is empty, since from then on no nodes are queried. Let τ_1 and τ_2 be random variables indicating the first step in which the frontier is empty for P_1 and P_2, respectively, starting from state S. Then

$$\mathbb{E}\left[b(S, P_1)\right] = \sum_{t=0}^{\tau_1 - 1} p_T e^{-\alpha T - \beta t}$$

$$\mathbb{E}\left[b(S, P_2)\right] = \sum_{t=0}^{\tau_2 - 1} p_T e^{-\alpha T - \beta t}$$

The order in which nodes are queried does not affect the first step in which the frontier is empty, so $\tau_1 \sim \tau_2$. Therefore $\mathbb{E}[b(S, P_1)] = \mathbb{E}[b(S, P_2)]$. □

DRAFT

Once $\alpha > \beta$, a less recent node has both a higher expected immediate benefit and more children in expectation, so querying nodes in order of reverse recency is optimal.

Theorem 6.5. *If $\alpha > \beta$, it is optimal to query nodes in order of reverse recency.*

Proof. Let σ be the optimal priority ordering constructed via the dynamic program in section A. It suffices to show that for all $0 \leq j \leq T$, $\sigma_j = T - j$.

Proof by induction. By definition,

$$\begin{aligned}\sigma_0 &= \operatorname*{arg\,max}_{h \in \{0,1,\ldots,T\}} p(h)e^{-\beta h} \\ &= \operatorname*{arg\,max}_{h \in \{0,1,\ldots,T\}} p_T e^{-\alpha T + (\alpha - \beta)h} \\ &= T\end{aligned}$$

Here T attains the maximum, since $\alpha > \beta$.

Fix $0 < k < T$. Assume that that for all $0 \leq j \leq k-1$, $\sigma_j = T - j$. As a result,

$$(\sigma_0, \sigma_1, \ldots, \sigma_{k-1}) = (T, T-1, \ldots, T-k+1)$$

Given this prefix, σ_k is selected by comparing (h, σ_{k-1})-periods for h not in the prefix, that is, for $h \in \{0, 1, \ldots, T-k\}$. Fix $h \in \{0, 1, \ldots, T-k\}$, and let v be a node with recency $h(v) = h$. Recall that an (h, σ_{k-1})-period consists of an $(h, \emptyset)$-period followed by a $(Z(h), \sigma_{k-1})$-epoch, for $Z(h) = (Z_0, Z_1, \ldots, Z_{h-1}) \sim D^h$. Since a node is less recent than its descendants, any descendant v' of v has recency $h(v') < h \leq T - k$, so the epoch only involves nodes with recencies in $\{0, 1, \ldots, T-k\}$. However, the prefix dictates that any node u queried during the epoch has recency $h(u) > T - k$. Thus no node is queried during the epoch, so for this particular prefix an (h, σ_{k-1})-period is equivalent to an $(h, \emptyset)$-period.

Therefore

$$\begin{aligned}\sigma_k &= \operatorname*{arg\,max}_{h \in \{0,1,\ldots,T-k\}} \frac{\mathbb{E}[b(h, \sigma_{k-1})]}{1 - \gamma(h, \sigma_{k-1})} \\ &= \operatorname*{arg\,max}_{h \in \{0,1,\ldots,T-k\}} \frac{\mathbb{E}[b(h, \emptyset)]}{1 - \gamma(h, \emptyset)} \\ &= \operatorname*{arg\,max}_{h \in \{0,1,\ldots,T-k\}} \frac{p_T e^{-\alpha T + (\alpha-\beta)h}}{1 - e^{-\beta}} \\ &= T - k\end{aligned}$$

Here $T - k$ attains the maximum, because $\alpha > \beta$. Thus for all $0 \leq j \leq T$, $\sigma_j = T - j$, so the optimal policy queries nodes in order of reverse recency. □

7. SI Appendix: Bivariate Model, Statement of Results

In the bivariate model the probability that a node is infected decays relative to the incubation period of its parent. Modeling this requires keeping track of two parameters, a node's recency and the recency of its parent. We say that a node of recency h with a parent of recency h' has *span* $\Delta = h' - h$. The probability that a node of span Δ is infected is $p(\Delta) = p_T e^{-\alpha\Delta}$. Aside from the probability of infection, the bivariate model is identical to the basic model, so a node's recency h determines the distribution on its children and the benefit returned if it is found to be infected. A node of recency h and span Δ is defined by its *type* (h, Δ), and policies in the bivariate model are on the set of types $\{0, 1, \ldots, T\}^2$. Thus the bivariate model demonstrates an analysis of policies that take into account multiple parameters. When $\alpha = 0$, the bivariate model instantiates the basic model.

Just as in the basic and univariate models we assume that nodes of the same recency are indistinguishable until they are queried, in the bivariate model we assume that nodes of the same type are indistinguishable until they are queried. Therefore we redefine the state S_t to be the multiset of types present in the frontier at time t. If the tracer queries an infected node, the query reveals the type of each contact. Recall that a node v of recency $h(v) = h$ has a multiset of children $Z(h) = (Z_0, Z_1, \ldots, Z_{h-1}) \sim D^h$, where Z_j indicates the number of children of recency j. Since $h(v) = h$, a child with recency j has span $i = h - j$. Therefore the children of v are defined by the multiset of types $Y(h) = (Y_{0,h}, Y_{1,h-1}, \ldots, Y_{h-1,1}) \sim D^h$, where $Y_{j,i}$ indicates the number of children with recency j and span i. Observe that $i + j = h$, which implies that nodes of different recencies have no children of the same type.

Our main result in this section is a monotonicity property that shows that it is optimal to query nodes of the same recency in order of increasing span.

Theorem 7.1. *In the bivariate model, there is an optimal policy that queries nodes with the same recency in order of increasing span.*

To prove this result, we analyze an optimal priority ordering using many of the same techniques developed in sections 4 and 6. The proof leverages the fact that nodes of the same recency have the same distribution on descendants in order to optimize over span.

As a complement to the main result, we also examine monotonicity along the dimension of recency. Since nodes of different recencies have no children of the same type, comparing periods becomes tricky, and the inductive approaches used in sections 4 and 6 do not seem to apply here. Instead we construct the optimal ordering of types step-by-step. To make this approach tractable, our result is restricted to settings where the contact distribution D is a Bernoulli distribution and where all nodes have recencies in $\{0, 1, 2\}$.

Theorem 7.2. *In the bivariate model, for any Bernoulli distribution D, a large enough constant $\beta > 0$, and restricted to types with recencies in $\{0, 1, 2\}$, it is optimal to query nodes of the same span in order of recency.*

While the other restrictions are due to our particular approach, a lower bound on β is in fact necessary; there are settings of β for which querying nodes of the same span in order of recency is not optimal. Full proofs of the above theorems are in section 9.

DRAFT

8. SI Appendix: General Model

The general model provides a broad framework that allows for a variety of factors to affect how individuals interact and how the infection spreads. In the general model each node is assigned an arbitrary type, which is associated with an arbitrary probability of infection and an arbitrary distribution on descendants. An individual's type could be thought of as representing information like their profession or role within a community and the context in which they are exposed. Thus the general model has the flexibility to describe the categorizations of contacts we see in practice, where the priority assigned to a contact depends on multiple different factors. The general model also encompasses the basic, univariate, and bivariate models.

We show in theorem 8.2 that the general model reduces to the branching bandit model. This has two main implications. First, it formally defines a connection between contact tracing and the branching bandit model. Second, it implies that the optimal policy for any instance of the general model can be found by analyzing the optimal policy for a corresponding instance of the branching bandit model, as constructed by the dynamic program in section A. We show in theorem 8.1 that, as a result, any instance of the general model has an optimal policy which is an efficiently computable index policy on the set of types with an efficient construction. Since the basic, univariate, and bivariate models are all instances of the general model, this result applies to these settings as well, and it defines the construction of optimal policies in each of the three models.

A. Model. The general model follows the same structure as the model from section 2, however the changes to the contact and infection processes warrant a second overview.

Phase 1 Phase 1 spans steps $t = -T$ through $t = 0$. During this phase each individual meets new contacts and infected individuals probabilistically infect each new contact they meet.

Each individual is associated with a known role $w \in \{1, 2, \dots, W\}$ and a hidden binary infection status $d \in \{0, 1\}$. Each individual also has a known recency $h \in \{0, 1, \dots, T\}$. An individual with recency h and role w belongs to category $(h, w) \in C$ where $C = \{1, 2, \dots, T\} \times \{1, 2, \dots, W\}$. After an individual is exposed, on each step t thereafter they meet new contacts defined by a multiset of categories drawn from $D_{c,d,t}$, a distribution on all multisets of elements in $[C]$. If the individual is infected, they infect each new contact they meet independently according to the function $p : [C] \times [C] \to [0, 1]$. The probability that an infected individual from category c' infects a contact in category c is $p(c', c)$. As in the model from section 2, we are agnostic to the origin of the index cases, and we model each index case as the child of an infected super-root $\perp$. An index case with category c is infected with probability $p(c(\perp), c)$.

Step $t = 0$. On step $t = 0$ the contact and infection processes halt and from then on no new infections occur.

To understand the system on step $t = 0$, consider an individual v from category $c(v) = c$. An individual in category c has recency $h(c)$, so v was exposed in step $-h(c)$. If v's exposure results in infection, for each step $-h(c) + 1 \le t \le 0$ in the remainder of Phase 1, v exposes $(Z_1(t), \dots, Z_C(t)) \sim D_{c,1,t}$ individuals, where $Z_j(t)$ indicates the number of individuals with category j exposed on step t. Then by the end of step $t = 0$, throughout the course of the first phase v has exposed a multiset of contacts $Z(c) = (Z_1, \dots, Z_C)$, where $Z_j = \sum_{t=-h(c)+1}^{0} Z_j(t)$. Let D_c be the distribution on $Z(c)$.

As in section 2, we can view v as a node in a tree of potential exposures, where the children of v are all the contacts v met after being exposed. Let u be the node which exposed v. Then with probability $p((c(u), c(v))$ node v is infected and has children with categories $(Z_1, \dots, Z_C) \sim D_c$.

Phase 2 Phase 2 begins on step $t = 0$ and continues indefinitely. During Phase 2 an individual's infection status is fixed but hidden. A contact tracer seeks to identify infected individuals as efficiently as possible.

As in section 2, contact tracing begins on step $t = 0$ when a set of index cases are identified. Initally the index cases are the only nodes available to query, and the tracer observes the category of each index case. From then on, at each step $t \ge 0$ the tracer selects one node from the frontier to query. Querying a node reveals its infection status, and if they are infected, its contacts are included in the frontier. The tracer may only query a node that is an index case or the contact of an infected node already queried. Querying an infected node v with category $c(v) = c$ at step t returns the benefit $b(c, t) = b(c)e^{-\beta t}$, for some arbitrary contant $b(c) \in [0, 1]$.

Now consider the information available to the tracer at each step. Any node v in the frontier has an infected parent u, where u is either the super-root or another node in the tree that was already queried. Thus the tracer knows both v's category $c(v)$ and u's category $c(u)$. As described above, these two parameters define the probability that v is infected, and if v is infected, the benefit of querying v and the distribution on children added to the frontier. That is, $c(u)$ and $c(v)$ fully define the distribution on outcomes that result from querying v. We say that v has *type* $(c(u), c(v)) \in [C]^2$. As in section 7, we assume two nodes of the same type are indistinguishable until they are queried. Then the state S_t is the multiset of types present in the frontier at time t, and at each step the tracer selects a node to query based on its type.

Defining the objective. On each step $t \ge 0$, the tracer selects a node v_t from the frontier to query where v_t has category $c(v_t)$. Since v_t is in the frontier, it was exposed to the infection. Let $\mathbb{1}(v_t)$ indicate whether v_t is infected (1) or uninfected (0). If v_t is infected, then benefit $b(c(v_t))e^{-\beta t}$ is returned. Thus the total benefit the tracer accumulates over the course of Phase 2 is

$$\sum_{t \ge 0} \mathbb{1}(v_t) \cdot b(c(v_t))e^{-\beta t}.$$

As in section 2, the objective is to develop a policy for querying nodes that maximizes the total expected benefit, where the expectation is taken over all realizations $\{\mathbb{1}(v_0), \mathbb{1}(v_1), \mathbb{1}(v_2), \dots\}$.

Defining the general model from types. The model can be equivalently described in terms of types instead of categories, which helps simplify the proof that follows. Enumerate the set of all types $[C]^2$ as $\{1, 2, \dots, C^2\}$. Let v be a node of type $(c', c) \in [C]^2$, where (c', c) is indexed as type $j \in \{1, 2, \dots, C^2\}$ in the enumeration. We now define the probability of infection, benefit, and distribution on children associated with type j. If v is exposed, the probability that v is infected is $\overline{p}(j) = p(c', c)$. If v is infected, the benefit of querying v is $\overline{b}(j) = b(c)$ and v's children have categories $(Z_1, \dots, Z_C) \sim D_c$, where Z_k indicates the number of children from category k. Since $c(v) = c$, any child of v from category k has type (c, k), so Z_k equivalently indicates the number of children of type $l = (c, k)$. Let $\overline{D}_j$ be the equivalent distribution on multisets of types in $\{1, 2, \dots, C^2\}$ so that for $(Y_1, \dots, Y_{C^2}) \sim \overline{D}_j$, Y_l indicates the number of children of type l. Then the state $S_t = (X_1, \dots, X_{C^2})$ is the multiset of types present in the frontier at time t.

DRAFT

B. Optimal policies in the general model. Our primary result shows that the optimal policy in the general model is an index policy on the set of types $\{1, 2, \ldots, C^2\}$.

Theorem 8.1. *For any instance of the general model, there is an optimal policy that is an index policy on the set of types. Moreover, this index policy has an efficient construction.*

Proof. Proof via reduction to the branching bandit model. Recall that the branching bandit model involves arms belonging to classes $\{1, \ldots, L\}$. When an arm of class i is pulled it yields a reward $R(i)$ and is replaced by a set of new arms $N_{i1}, \ldots, N_{iL}$, where each class i has an arbitrary, known, joint distribution on the random variables $R(i)$ and $N_{i1}, \ldots, N_{iL}$. Additionally, each class i is also associated with a random variable $\mu(i)$, where pulling an arm from class i occupies $\mu(i)$ steps, however for the purposes of this reduction we consider only a restricted model where each pull occupies exactly 1 step. At step t the system is defined by a vector $n(t) = (N_1, \ldots, N_L)$, where N_i is the total number of arms of class i available, and a reward received at step t is discounted by $e^{-\eta t}$ for a fixed parameter $\eta > 0$. As described in section A, the optimal policy in the branching bandit model is an index policy on the set of classes $\{1, \ldots, L\}$ with an efficient construction.

The plan for the reduction is to map each node to an arm. The idea is to map the benefit and new children returned by querying a node to the reward and new arms returned by pulling an arm. To do this, for each type in the contact tracing instance we construct a class in the branching bandit instance with an appropriate distribution on reward and new arms. Theorem 8.2 proves that, for a specific mapping from types to classes, the general model reduces to the branching bandit model. Informally, proving the reduction involves showing that querying a sequence of nodes maps to pulling a corresponding sequence of arms.

The claim follows from this reduction. Since types map to classes, at any step t the state of the frontier S_t is represented by a vector of arms $n(t)$. The optimal policy in the branching bandit model selects the next arm to pull from $n(t)$, which by the reduction dictates the next node to query from the frontier. Thus the optimal policy in the branching bandit model defines the optimal policy in the general model. In particular, since the former is an index policy on the set of classes, the latter is an index policy on the set of types. Therefore the optimal policy in the general model is an index policy on the set of types with an efficient construction. □

DRAFT

Before continuing with the reduction, we first describe what proving such a reduction requires. We start by defining the general model and the branching bandit model each as games where an agent chooses actions in order to transition between different states. (Here "state" is used in the general sense of an agent moving through different states in a game, not as a multiset of types.) Proving a reduction involves mapping a sequence of states and actions in the general model to a corresponding sequence of states and actions in the branching bandit model.

In the general model, the tracer (the agent) chooses types (actions) and in the branching bandit model the agent chooses classes (actions). Specifically, in the general model the state at step t is a pair (S_t, b_t) where b_t is the total benefit accumulated through the start of step t. The tracer chooses a type $j_t \in S_t$, and in response a random benefit $B(j_t)e^{-\beta t}$ is returned and a multiset of new children $(Y_1, \ldots, Y_{C^2}) \sim D_{j_t}$ is added to the frontier. As a result, the tracer transitions to the state (S_{t+1}, b_{t+1}) where $S_{t+1} = (S_t \backslash j_t) \cup (Y_1, \ldots, Y_{C^2})$ and $b_{t+1} = b_t + B(j_t)e^{-\beta t}$. In the branching bandit model, the state at step t is a pair $(n(t), r_t)$ where r_t is the total reward earned through the start of step t. The agent chooses a class $i_t \in n(t)$, and in response a random reward $R(i_t)e^{-\eta t}$ is received and a multiset of new arms $(N_{i_t 1}, \ldots, N_{i_t L})$ are added to the system. As a result, the agent transitions to the state $(n(t+1), r_{t+1})$ where $n(t+1) = (n(t) \setminus i_t) \cup (N_{i_t 1}, \ldots, N_{i_t L})$ and $r_{t+1} = r_t + R(i_t)e^{-\eta t}$.

Let (S_0, b_0) be an arbitrary initial state, and let $(S_0, b_0, j_0), \ldots, (S_{t'}, b_{t'}, j_{t'})$ be a sequence of states and actions. The general model reduces to the branching bandit model if there is a mapping of states and actions such that the next-state distribution is preserved. Specifically, we need a mapping from types to classes so that a corresponding sequence $(n(0), r_0, i_0), \ldots, (n(t'), r_{t'}, i_{t'})$ exists which fulfills the following criteria for all $0 \leq t \leq t'$:

(1) S_t maps to $n(t)$, $b_t = r_t$, and j_t maps to i_t.

(2) $(S_{t+1}, b_{t+1}) \mid (S_t, b_t, j_t)$ has the same distribution as $(n(t+1), r_{t+1}) \mid (n(t), r_t, i_t)$.

If we can establish both (1) and (2) then for any sequence of states and actions the distribution on outcomes after some number of steps k is the same in both models. The following theorem proves the reduction by constructing a mapping from types to classes that satisfies these criteria.

Theorem 8.2. *The general model reduces to the branching bandit model.*

Proof. Let $L = C^2$ and let $\eta = \beta$. For each type $j \in \{1, 2, \ldots, C^2\}$ we define a class $i = j$. (Even though $i = j$, we use separate names to distinguish between the class and type.) By this mapping, for a state $S_t = (X_1, \ldots, X_{C^2})$ and vector $N(t) = (N_1, \ldots, N_L)$, if $(X_1, \ldots, X_{C^2}) = (N_1, \ldots, N_L)$ then S_t maps to $N(t)$. First we define the mapping, and then we show the reduction.

The idea for the mapping is to construct a class i for each type j such that the joint distribution on reward and new arms associated with i maps to the joint distribution on benefit and new children associated with j.

Recall that the joint distribution on the benefit and multiset of children associated with type j is

$$(B(j), (Y_{j1}, \ldots, Y_{jC^2})) = \begin{cases} \text{w.p. } \overline{p}(j) & (\bar{b}(j), (U_{j1}, \ldots, U_{jC^2}) \sim \overline{D}_j) \\ \text{w.p. } 1 - \overline{p}(j) & (0, \emptyset) \end{cases}$$

While so far we have referred to $\overline{D}_j$ as a distribution on multisets of types, $\overline{D}_j$ is simply a distribution on multisets of elements in $\{1, 2, \ldots, C^2\}$. Since $L = C^2$, it can also be viewed as a distribution on multisets of classes $\{1, 2, \ldots, L\}$. We define class i by the joint distribution

$$(R(i),(N_{i1},\dots,N_{iL})) = \begin{cases} \text{w.p. } \overline{p}(j) & (\overline{b}(j),(M_{j1},\dots,M_{jL}) \sim \overline{D}_j) \\ \text{w.p. } 1-\overline{p}(j) & (0,\emptyset) \end{cases}$$

By this construction, if $i = j$ then $(B(j),(Y_{j1},\dots,Y_{jC^2})) \sim (R(i),(N_{i1},\dots,N_{iL}))$.

For any $t \geq 0$, let (S_t, b_t) be an arbitrary state in the general model, and suppose the tracer chooses type $j \in S_t$. Then $b_{t+1} = b_t + B(j)e^{-\beta t}$ and $S_{t+1} = (S_t \setminus j) \cup (Y_{j1},\dots,Y_{jC^2})$. Let $(n(t), r_t) = (S_t, b_t)$ be the corresponding state in the branching bandit model, and let the agent choose the corresponding class $i = j$. Then $r_{t+1} = r_t + R(i)e^{-\eta t}$ and $n(t+1) = (n(t) \setminus i) \cup (N_{i1},\dots,N_{iL})$. Because $i = j$, $(B(j),(Y_{j1},\dots,Y_{jC^2})) \sim (R(i),(N_{i1},\dots,N_{iL}))$, and combined with the fact that $n(t) = S_t$, $\eta = \beta$, and $r_t = b_t$,

$$\begin{aligned}(S_{t+1}, b_{t+1}) \mid (S_t, b_t, j) &\sim ((S_t \setminus j) \cup (Y_{j1},\dots,Y_{jC^2}), b_t + B(j)e^{-\beta t}) \mid (S_t, b_t, j) \\ &\sim ((n(t) \setminus i) \cup (N_{i1},\dots,N_{iL}), r_t + R(i)e^{-\eta t}) \mid (n(t), r_t, i) \\ &\sim (n(t+1), r_{t+1}) \mid (n(t), r_t, i)\end{aligned}$$

Therefore, given corresponding states $(S_t, b_t) = (n(t), r_t)$ and actions $j_t = i_t$, then

$$(S_{t+1}, b_{t+1}) \mid (S_t, b_t, j_t) \sim (n(t+1), r_{t+1}) \mid (n(t), r_t, i_t).$$

Since the mapping preserves this next-state transition, by induction there is a sequence of corresponding states and actions in the branching bandit model, so the reduction holds. □

9. SI Appendix: Bivariate Model, Proofs

Recall that each node in the bivariate model is associated with a recency $h \in \{0, 1, \dots, T\}$ and span $\Delta \in \{0, 1, \dots, T\}$, summarized by a type $(h, \Delta) \in \{0, 1, \dots, T\}^2$. A node of span Δ has probability of infection $p(\Delta) = p_T e^{-\alpha\Delta}$. Policies in the bivariate model are on the set of all types $\{0, 1, \dots, T\}^2$.

Defining the optimal policy. Recall that the bivariate model is identical to the basic model except for the probability of infection. Therefore we can construct the optimal policy by modifying the dynamic program from section B. The element σ_0 is assigned to the type with the highest expected immediate benefit.

$$\sigma_0 = \underset{(h,\Delta)\in\{0,1,\dots,T\}^2}{\arg\max} \mathbb{E}[b((h,\Delta),\emptyset)] \qquad [16]$$

Given the first k elements $\sigma_0, \sigma_1, \dots, \sigma_{k-1}$, σ_k is selected by comparing $((h,\Delta), \sigma_{k-1})$-periods across all types not already in the prefix.

$$\sigma_k = \underset{(h,\Delta)\in\{0,1,\dots,T\}^2\setminus\{\sigma_0,\dots,\sigma_{k-1}\}}{\arg\max} \frac{\mathbb{E}[b((h,\Delta),\sigma_{k-1})]}{1-\gamma((h,\Delta),\sigma_{k-1})} \qquad [17]$$

Analyzing periods. Recall that a node with recency h has children $Y(h) = (Y_{0,h}, Y_{1,h-1}, \dots, Y_{h-1,1}) \sim D^h$, where $Y_{j,i}$ indicates the number of children with recency j and span i. For any prefix $\sigma_0, \sigma_1, \dots, \sigma_{k-1}$, an $((h,\Delta),\sigma_{k-1})$-period consists of an $((h,\Delta),\emptyset)$-period followed by a $(Y(h),\sigma_{k-1})$-epoch for $Y(h) \sim D^h$. Therefore,

$$\mathbb{E}[b((h,\Delta),\sigma_{k-1})] = p(\Delta)\left(e^{-\beta h} + e^{-\beta} \underset{Y(h)\sim D^h}{\mathbb{E}}[b(Y(h),\sigma_{k-1})]\right) \qquad [18]$$

$$\gamma((h,\Delta),\sigma_{k-1}) = e^{-\beta}\left(p(\Delta) \underset{Y(h)\sim D^h}{\mathbb{E}}\left[e^{-\beta\tau(Y(h),\sigma_{k-1})}\right] + 1 - p(\Delta)\right) \qquad [19]$$

Restating the optimal policy. We restate the optimal policy using the above identities.

$$\begin{aligned}\sigma_0 &= \underset{(h,\Delta)\in\{0,1,\dots,T\}^2}{\arg\max} \mathbb{E}[b((h,\Delta),\emptyset)] \\ &= \underset{(h,\Delta)\in\{0,1,\dots,T\}^2}{\arg\max} p(\Delta)e^{-\beta h}\end{aligned} \qquad [20]$$

Given the first k elements $\sigma_0, \sigma_1, \dots, \sigma_{k-1}$, σ_k is selected from the elements not already in the prefix.

$$\begin{aligned}\sigma_k &= \underset{(h,\Delta)\in\{0,1,\dots,T\}^2\setminus\{\sigma_0,\dots,\sigma_{k-1}\}}{\arg\max} \frac{\mathbb{E}[b((h,\Delta),\sigma_{k-1})]}{1-\gamma((h,\Delta),\sigma_{k-1})} \\ &= \underset{(h,\Delta)\in\{0,1,\dots,T\}^2\setminus\{\sigma_0,\dots,\sigma_{k-1}\}}{\arg\max} \frac{p(\Delta)\left(e^{-\beta h} + e^{-\beta}\,\mathbb{E}_{Y(h)\sim D^h}[b(Y(h),\sigma_{k-1})]\right)}{1 - e^{-\beta}\left(p(\Delta)\,\mathbb{E}_{Y(h)\sim D^h}\left[e^{-\beta\tau(Y(h),\sigma_{k-1})}\right] + 1 - p(\Delta)\right)}\end{aligned} \qquad [21]$$

A. Ordering types of the same recency. Using similar techniques as in section 6, we present our main result for the bivariate section.

Theorem 8.1. *In the bivariate model, there is an optimal policy that queries nodes with the same recency in order of increasing span.*

DRAFT

Proof. Fix $T \in \mathbb{N}$, $p_T \in (0, 1]$, and $\beta > 0$. Let σ be the optimal priority ordering constructed via the dynamic program described above. Fix $h \in \{0, 1, \ldots, T\}$. It suffices to show that $(h, 0) \preceq (h, 1) \preceq \cdots \preceq (h, T)$.

By eq. (20)

$$\begin{aligned}\sigma_0 &= \operatorname*{arg\,max}_{(h,\Delta)\in\{0,1,\ldots,T\}^2} \mathbb{E}[b(h, \emptyset)] \\ &= \operatorname*{arg\,max}_{(h,\Delta)\in\{0,1,\ldots,T\}^2} p_T e^{-\alpha\Delta-\beta h} \\ &= (0, 0)\end{aligned}$$

Since $\alpha > 0$ and $\beta > 0$, $(0, 0)$ attains the maximum. It suffices to show that for any $(h, \Delta_1) \neq (0, 0)$ and $(h, \Delta_2) \neq (0, 0)$ if $\Delta_1 < \Delta_2$ then $(h, \Delta_1) \prec (h, \Delta_2)$, since then by induction $(h, 0) \preceq (h, 1) \preceq \cdots \preceq (h, T)$.

Fix any $(h, \Delta_1) \neq (0, 0)$ and $(h, \Delta_2) \neq (0, 0)$. Assume to the contrary that $(h, \Delta_2) \prec (h, \Delta_1)$. Then (h, Δ_2) was selected prior to (h, Δ_1) in the construction of σ. That is, there is some pair k, k' with $k < k'$ such that $\sigma_k = (h, \Delta_2)$ and $\sigma_{k'} = (h, \Delta_1)$. As a result, for the prefix $\sigma_0, \ldots, \sigma_{k-1}$,

$$\frac{\mathbb{E}[b((h, \Delta_2), \sigma_{k-1})]}{1 - \gamma((h, \Delta_2), \sigma_{k-1})} \geq \frac{\mathbb{E}[b((h, \Delta_1), \sigma_{k-1})]}{1 - \gamma((h, \Delta_1), \sigma_{k-1})}$$

Given the prefix $\sigma_0, \sigma_1, \ldots, \sigma_{k-1}$, define the expected benefit and pre-multiplier associated with a $(Y(h), \sigma_{k-1})$-epoch for $Y(h) \sim D^h$.

$$\begin{aligned}b_h &= \mathop{\mathbb{E}}_{Y(h)\sim D^h}[b(Y(h), \sigma_{k-1})] \\ \gamma_h &= \mathop{\mathbb{E}}_{Y(h)\sim D^h}[\gamma(Y(h), \sigma_{k-1})]\end{aligned}$$

Applying these identities to eqs. (18) and (19), for any $\Delta \in \{0, 1, \ldots, T\}$,

$$\frac{\mathbb{E}[b((h, \Delta), \sigma_{k-1})]}{1 - \gamma((h, \Delta), \sigma_{k-1})} = \frac{p_T e^{-\alpha\Delta}\left(e^{-\beta h} + e^{-\beta} b_h\right)}{1 - e^{-\beta} + e^{-\beta}(1 - \gamma_h) p_T e^{-\alpha\Delta}}$$

Define the function $f_h : \mathbb{R} \to \mathbb{R}$ as

$$f_h(x) = \frac{p_T e^{-\alpha x}\left(e^{-\beta h} + e^{-\beta} b_h\right)}{1 - e^{-\beta} + e^{-\beta}(1 - \gamma_h) p_T e^{-\alpha x}}$$

Then

$$\begin{aligned}f_h(\Delta_1) &= \frac{\mathbb{E}[b((h, \Delta_1), \sigma_{k-1})]}{1 - \gamma((h, \Delta_1), \sigma_{k-1})} \\ f_h(\Delta_2) &= \frac{\mathbb{E}[b((h, \Delta_2), \sigma_{k-1})]}{1 - \gamma((h, \Delta_2), \sigma_{k-1})}\end{aligned}$$

Since $\sigma_k = (h, \Delta_2)$, $f_h(\Delta_2) \geq f_h(\Delta_1)$. For strictly positive constants

$$\begin{aligned}C_0 &= p_T\left(e^{-\beta h} + e^{-\beta} b_h\right) \\ C_1 &= 1 - e^{-\beta} \\ C_2 &= p_T e^{-\beta}(1 - \gamma_h),\end{aligned}$$

we can rewrite f_h as

$$f_h(x) = \frac{C_0 e^{-\alpha x}}{C_1 + C_2 e^{-\alpha x}}$$

Then

$$f_h'(x) = \frac{-C_0 C_1 \alpha e^{-\alpha x}}{(C_1 + C_2 e^{-\alpha x})^2}$$

Since $C_0, C_1, C_2 > 0$, $f_h'(x) < 0$ for all $x \in \mathbb{R}$. Therefore f_h is monotonically decreasing. This implies $f_h(\Delta_2) < f_h(\Delta_1)$, which contradicts the assumption that $f_h(\Delta_2) \geq f_h(\Delta_1)$. Thus no such prefix $\sigma_0, \sigma_1, \ldots, \sigma_{k-1}$ exists, so $(h, \Delta_1) \preceq (h, \Delta_2)$, and as a result $(h, 0) \preceq (h, 1) \preceq \cdots \preceq (h, T)$. ☐

B. Ordering types of the same span. In this section we examine ordering types with the same span. Recall that two nodes of different recencies have no children of the same type. This makes comparing periods and epochs tricky, and as a result, this section uses fairly different techniques. In order to determine whether $(h, \Delta) \prec (h', \Delta')$, we analyze the construction of optimal policies step by step and examine which type appears first in the ordering. Our techniques require case-by-case analysis, so we restrict our analysis to the setting in which D is a Bernoulli distribution and the recencies of all nodes are limited to $\{0, 1, 2\}$.

Our main result shows that, under these restrictions, it is optimal to query nodes of the same span in order of recency.

Theorem 8.2. *In the bivariate model, for any Bernoulli distribution D, a large enough constant $\beta > 0$, and restricted to types with recencies in $\{0, 1, 2\}$, it is optimal to query nodes of the same span in order of recency.*

Proof. Fix $T \in \mathbb{N}$, $p_T \in (0, 1]$, $c \in (0, 1]$, $\alpha > 0$, and $\beta > \ln\left(2(1 + c p_T e^{-\alpha})/(1 - e^{-\alpha})\right)$. Let $D = \mathrm{Ber}(c)$. It suffices to show that there is an optimal policy where for any $\Delta \in \{0, 1, \ldots, T\}$, $(0, \Delta) \preceq (1, \Delta) \preceq (2, \Delta)$. Lemma 10.1 shows that $(0, \Delta) \preceq (1, \Delta)$, and lemma 10.2 shows that $(1, \Delta) \preceq (2, \Delta)$, giving the desired result. ☐

DRAFT

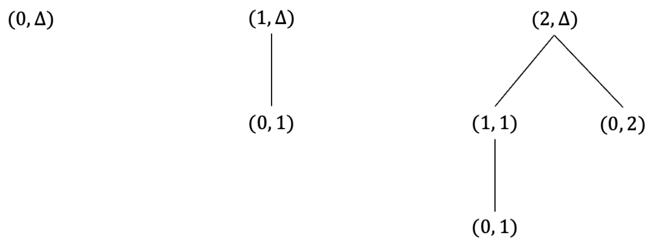

Fig. 3. In comparing types with the same span Δ, we examine a version of the bivariate model where each individual meets at most one contact each day. A node of recency 0 has no potential children, a node of recency 1 has one potential child, and a node of recency 2 has two potential children, one with a potential child of its own.

Overview of techniques. Recall that a node of type (h, Δ) has children defined by the multiset $Y(h) = (Y_{0,h}, Y_{1,h-1}, \ldots, Y_{h-1,1}) \sim D^h$, where $Y_{j,i}$ indicates the number of children with recency j and span i. For the following proofs D is a Bernoulli distribution, so $Y_{j,i} \in \{0, 1\}$. We say that a node v with recency $h(v) = h$ has *potential children* $\{(0, h), (1, h-1), \ldots, (h-1, 1)\}$, since each type in the set could be a child of v. Let $W(h, \Delta)$ be the set of all *potential descendants* of a node of type (h, Δ). Figure 3 shows the potential descendants of different types we examine in this section.

Our strategy is to analyze the dynamic program from eqs. (16) and (17). By definition, an optimal priority ordering σ sequences all types in $\{0, 1, \ldots, T\}^2$. However, determining whether $(h, \Delta) \preceq (h', \Delta')$ does not necessarily require computing the entirety of σ. Instead, we only need to determine which of (h, Δ) and (h', Δ') falls earlier in the ordering σ. To do this, for any prefix $\sigma_0, \sigma_1, \ldots, \sigma_{k-1}$, we need to be able to compare an $((h, \Delta), \sigma_{k-1})$-period to an $((h', \Delta'), \sigma_{k-1})$-period. An $((h, \Delta), \sigma_{k-1})$-period only involves the type (h, Δ) and its potential descendants. Therefore, to decide whether $(h, \Delta) \preceq (h', \Delta')$, it is sufficient to compute an optimal ordering on only the two types in question, (h, Δ) and (h', Δ'), and their potential descendants, $W(h, \Delta)$ and $W(h', \Delta')$. Specifically, we construct an ordering π on the set of types

$$S_\pi = (h, \Delta) \cup (h', \Delta') \cup W(h, \Delta) \cup W(h', \Delta')$$

such that if $(h, \Delta) \preceq (h', \Delta')$ according to π, it follows that there is an optimal ordering σ on $\{0, 1, \ldots, T\}^2$ such that π is a subsequence of σ and therefore $(h, \Delta) \preceq (h', \Delta')$ according to σ.

To simplify notation, for any type (h, Δ) and prefix $\pi_0, \pi_1, \ldots, \pi_{k-1}$, we define the index function

$$I((h, \Delta), \pi_{k-1}) = \frac{\mathbb{E}[b((h, \Delta), \pi_{k-1})]}{1 - \gamma((h, \Delta), \pi_{k-1})}$$

Overloading notation, for any recency $h \in \{0, 1, \ldots, T\}$ we define the benefit function

$$b(h) = e^{-\beta h}$$

The dynamic program is defined exactly as before, now with the index function notation. The highest priority element according to π is the type in S_π with the highest expected immediate benefit.

$$\pi_0 = \underset{(h,\Delta) \in S_\pi}{\arg\max}\ I((h, \Delta), \emptyset)$$

Given a prefix $\pi_0, \pi_1, \ldots, \pi_{k-1}$, π_k is chosen from the remaining elements in S_π.

$$\pi_k = \underset{(h,\Delta) \in S_\pi \setminus \{\pi_0, \ldots, \pi_{k-1}\}}{\arg\max} I((h, \Delta), \pi_{k-1})$$

Proof organization. The proof is split into two main lemmas. Lemma 10.1 shows that $(0, \Delta) \preceq (1, \Delta)$, and lemma 10.2 shows that $(1, \Delta) \preceq (2, \Delta)$. Lemma 10.1 serves as an introduction to constructing optimal priority orderings in a fairly simple setting where π involves only three types, and lemma 10.2 extends these techniques to handle a larger set of types. Because of this added complexity, the proof of lemma 10.2 is split into cases. To show that $(1, \Delta) \preceq (2, \Delta)$, lemmas 10.3 and 10.4 handle the cases when $\Delta \in \{0, 1\}$ and lemmas 10.6–10.8 handle the cases when $\Delta > 1$.

B.1. Proving $(0, \Delta) \preceq (1, \Delta)$.

Lemma 10.1. *Fix $T \in \mathbb{N}$, $\Delta \in \{0, 1, \ldots, T\}$, $p_T \in (0, 1]$, $c \in (0, 1]$, $\alpha > 0$, and $\beta > \ln(1 + cp_T e^{-\alpha} - cp_T e^{-\alpha\Delta})$. Let $D = \mathrm{Ber}(c)$. Then $(0, \Delta) \preceq (1, \Delta)$.*

Proof. Recall that our strategy is to construct an ordering π that is a subsequence of an optimal ordering σ. Consider $\Delta = 0$. Then $S_\pi = \{(0, 0), (1, 0), (0, 1)\}$, so

$$\pi_0 = \underset{(h,\Delta) \in S_\pi}{\arg\max}\ I((h, \Delta), \emptyset) = \underset{(h,\Delta) \in S_\pi}{\arg\max} \frac{p_T e^{-\alpha\Delta - \beta h}}{1 - e^{-\beta}} = (0, 0)$$

Thus $(0, 0) \prec (0, 1)$.

Consider $\Delta = 1$. Then $S_\pi = \{(0, 1), (1, 1)\}$, so

$$\pi_0 = \underset{(h,\Delta) \in S_\pi}{\arg\max}\ I((h, \Delta), \emptyset) = \underset{(h,\Delta) \in S_\pi}{\arg\max} \frac{p_T e^{-\alpha\Delta - \beta h}}{1 - e^{-\beta}} = (0, 1)$$

Thus $(0, 1) \prec (1, 1)$.

DRAFT

Now consider $\Delta > 1$. Then $S_\pi = \{(0,\Delta),(1,\Delta),(0,1)\}$, so

$$\pi_0 = \underset{(h,\Delta)\in S_\pi}{\arg\max}\; I((h,\Delta),\emptyset) = \underset{(h,\Delta)\in S_\pi}{\arg\max}\; \frac{p_T e^{-\alpha\Delta-\beta h}}{1-e^{-\beta}} = (0,1)$$

Here, since $\Delta > 1$, $(0,1)$ maximizes the index function. To select π_1 we compare $I((0,\Delta),\pi_0)$ and $I((1,\Delta),\pi_0)$. The type $(0,\Delta)$ has no potential children, so

$$\begin{aligned} I((0,\Delta),\pi_0) &= I((0,\Delta),\emptyset) \\ &= \frac{p(\Delta)b(0)}{1-e^{-\beta}} \end{aligned} \qquad [22]$$

The type $(1,\Delta)$ has potential child $(0,1)$. A $((1,\Delta),\pi_0)$-period begins by querying a node of type $(1,\Delta)$ on step $t=0$. With probability $p(\Delta)$, the node is infected, and with probability c it has child $(0,1)$. So with probability $cp(\Delta)$ the child $(0,1)$ is queried on step $t=1$, otherwise the period ends on step $t=0$. Therefore

$$\begin{aligned} I((1,\Delta),\pi_0) &= \frac{\mathbb{E}[b((1,\Delta),\pi_0)]}{1-\gamma((1,\Delta),\pi_0)} \\ &= \frac{p(\Delta)(b(1)+cp(1)b(0)e^{-\beta})}{1-[(1-cp(\Delta))e^{-\beta}+cp(\Delta)e^{-2\beta}]} \\ &= \frac{p(\Delta)(b(1)+cp(1)b(0)e^{-\beta})}{1-e^{-\beta}+cp(\Delta)e^{-\beta}-cp(\Delta)e^{-2\beta}} \\ &= \frac{p(\Delta)(b(1)+cp(1)b(0)e^{-\beta})}{(1-e^{-\beta})(1+cp(\Delta)e^{-\beta})} \end{aligned} \qquad [23]$$

Combining eqs. (22) and (23) we have

$$\begin{aligned} I((0,\Delta),\pi_0) - I((1,\Delta),\pi_0) &= \frac{p(\Delta)b(0)}{1-e^{-\beta}} - \frac{p(\Delta)(b(1)+cp(1)b(0)e^{-\beta})}{(1-e^{-\beta})(1+cp(\Delta)e^{-\beta})} \\ &= \frac{p(\Delta)}{1-e^{-\beta}}\cdot\left(b(0) - \frac{b(1)+cp(1)b(0)e^{-\beta}}{1+cp(\Delta)e^{-\beta}}\right) \\ &= \frac{p_T e^{-\alpha\Delta}}{1-e^{-\beta}}\cdot\left(1-\frac{e^{-\beta}+cp_T e^{-\alpha-\beta}}{1+cp_T e^{-\alpha\Delta-\beta}}\right) \end{aligned}$$

The above expression is strictly positive iff

$$1 > \frac{e^{-\beta}+cp_T e^{-\alpha-\beta}}{1+cp_T e^{-\alpha\Delta-\beta}}$$

Setting $\beta > \ln(1+cp_T e^{-\alpha} - cp_T e^{-\alpha\Delta})$ satisfies the above inequality, so $I((0,\Delta),\pi_0) > I((1,\Delta),\pi_0)$. Thus $\pi_1 = (0,\Delta)$, and thus for $\Delta > 1$, $(0,\Delta) \prec (1,\Delta)$. Therefore for any $\Delta \in \{0,1,\dots,T\}$, $(0,\Delta) \prec (1,\Delta)$. ☐

DRAFT

B.2. Proving $(1,\Delta) \preceq (2,\Delta)$**.** The following lemma analyzes an optimal priority ordering case by case to show that in all cases for the given regime, $(1,\Delta) \preceq (2,\Delta)$.

Lemma 10.2. *Fix $T\in\mathbb{N}$, $\Delta\in\{0,1,\dots,T\}$, $p_T\in(0,1]$, $c\in(0,1]$, $\alpha>0$, and $\beta > \ln\left(2(1+cp_Te^{-\alpha})/(1-e^{-\alpha})\right)$. Let $D = \mathrm{Ber}(c)$. Then $(1,\Delta)\preceq(2,\Delta)$.*

Proof. Recall that the proof idea is to construct an ordering π on a subset of types, where π is a subsequence of an optimal ordering σ on $\{0,1,\dots,T\}^2$. Specifically, let $S_\pi = \{(0,1),(1,1),(0,2),(1,\Delta),(2,\Delta)\}$. We will analyze a priority ordering π on S_π. The cases for $\Delta=0$ and $\Delta=1$ are covered by lemma 10.3 and lemma 10.4, respectively. We analyze the case when $\Delta>1$ in multiple parts, as follows.

Fix $\Delta\in\{2,\dots,T\}$. Then, as in lemmas 10.1, 10.3 and 10.4, $\pi_0=(0,1)$. Next we examine π_1. By theorem 8.1 $(1,1)\preceq(1,\Delta)$, and by lemma 10.4 and theorem 8.1 $(1,1)\preceq(2,1)\preceq(2,\Delta)$. Therefore neither $(1,\Delta)$ nor $(2,\Delta)$ can be assigned to π_1, leaving $(1,1)$ and $(0,2)$ as the only remaining candidates. We now examine both cases and show that regardless of whether $\pi_1=(1,1)$ or $\pi_1=(0,2)$, $(1,\Delta)\preceq(2,\Delta)$.

Suppose $\pi_1=(0,2)$. Consider selecting π_2 from the set of remaining types $\{(1,1),(1,\Delta),(2,\Delta)\}$. Following the same argument as described above, $(1,1)$ precedes both $(1,\Delta)$ and $(2,\Delta)$, so $\pi_2=(1,1)$. For the given prefix, by lemma 10.6 $I((1,\Delta),\pi_2) > I((2,\Delta),\pi_2)$ so $\pi_3=(1,\Delta)$, and therefore $(1,\Delta)\preceq(2,\Delta)$.

Suppose $\pi_1=(1,1)$. Consider selecting π_2 from the set of remaining types $\{(0,2),(1,\Delta),(2,\Delta)\}$. By lemma 10.7 $I((1,\Delta),\pi_1) > I((2,\Delta),\pi_1)$, so $\pi_2\neq(2,\Delta)$. If $\pi_2=(1,\Delta)$, then $(1,\Delta)\preceq(2,\Delta)$. Suppose instead that $\pi_2=(0,2)$. For the given prefix, by lemma 10.8 $I((1,\Delta),\pi_2) > I((2,\Delta),\pi_2)$, so $\pi_3=(1,\Delta)$. Thus with the given bound on β, in any optimal priority ordering $(1,\Delta)\preceq(2,\Delta)$. ☐

Lemmas 10.3 and 10.4 follow similar structures, and the relatively minor differences between the two proofs are due to the change in span.

Lemma 10.3. *Fix $T\in\mathbb{N}$, $p_T\in(0,1]$, $c\in(0,1]$, and $\alpha,\beta>0$. Let $D=\mathrm{Ber}(c)$. Then $(1,0)\preceq(2,0)$.*

Proof. Let $S_\pi = \{(0,1),(1,0),(1,1),(0,2),(2,0)\}$. Since $(1,0)$ and $(1,1)$ both have recency 1, by theorem 8.1 it is optimal to query $(1,0)$ before $(1,1)$. Therefore, there is an optimal ordering σ where $(1,0) \preceq (1,1)$. Then for some pair k, k', with $k' > k$, $\pi_k = (1,0)$ and $\pi_{k'} = (1,1)$. Since $(1,1) \notin \{\pi_0, \dots, \pi_k\}$, when computing $I((2,0),\pi_j)$ for any $0 \le j \le k$ the potential child $(1,1)$ is never queried. Therefore we can ignore the potential child $(1,1)$ when computing π, since it does not affect which of $(1,0)$ or $(2,0)$ appears first. Instead, for the purposes of this particular proof, we can think of $(2,0)$ as having a single potential child $(0,2)$. Thus we only need to choose an ordering on the types $S'_\pi = \{(0,1),(1,0),(0,2),(2,0)\}$.

Consider $\alpha \ge \beta$. Then the type $(1,0)$ (which recall has recency $h = 1$ and span $\Delta = 0$) is assigned to π_0.

$$\pi_0 = \operatorname*{arg\,max}_{(h,\Delta)\in S'_\pi} I((h,\Delta),\emptyset) = \operatorname*{arg\,max}_{(h,\Delta)\in S'_\pi} \frac{p_T e^{-\alpha\Delta-\beta h}}{1-e^{-\beta}} = (1,0)$$

Thus if $\alpha \ge \beta$, $(1,0) \preceq (2,0)$.

Consider $\alpha < \beta$. Then

$$\pi_0 = \operatorname*{arg\,max}_{(h,\Delta)\in S'_\pi} I((h,\Delta),\emptyset) = \operatorname*{arg\,max}_{(h,\Delta)\in S'_\pi} \frac{p_T e^{-\alpha\Delta-\beta h}}{1-e^{-\beta}} = (0,1)$$

Given the prefix π_0, we now compute π_1. The possible candidates for π_1 are $(1,0)$, $(2,0)$ and $(0,2)$. Since neither $(2,0)$ nor $(0,2)$ has π_0 as a child, $I((2,0),\pi_0) = I((2,0),\emptyset)$ and $I((0,2),\emptyset) = I((0,2),\emptyset)$. Since $\alpha < \beta$,

$$I((0,2),\pi_0) = I((0,2),\emptyset) > I((2,0),\pi_0) = I((2,0),\emptyset)$$

Therefore $\pi_1 \ne (2,0)$, leaving $(1,0)$ and $(0,2)$ as the remaining candidates. If $\pi_1 = (1,0)$, then $(1,0) \preceq (2,0)$, and we are done. Suppose $\pi_1 = (0,2)$. Now we compute π_2 by comparing $I((1,0),\pi_1)$ and $I((2,0),\pi_1)$. The type $(1,0)$ has potential child $(0,1)$ in the prefix and the type $(2,0)$ has potential child $(0,2)$ in the prefix. Mirroring the process described in lemma 10.1,

$$\begin{aligned}
I((1,0),\pi_1) &= \frac{\mathbb{E}[b((1,0),\pi_1)]}{1-\gamma((1,0),\pi_1)} \\
&= \frac{p(0)(b(1)+cp(1)b(0)e^{-\beta})}{1-[(1-cp(0))e^{-\beta}+cp(\Delta)e^{-2\beta}]} \\
&= \frac{p(0)(b(1)+cp(1)b(0)e^{-\beta})}{1-e^{-\beta}+cp(0)e^{-\beta}-cp(0)e^{-2\beta}} \\
&= \frac{p(0)(b(1)+cp(1)b(0)e^{-\beta})}{(1-e^{-\beta})(1+cp(0)e^{-\beta})}
\end{aligned}$$

$$\begin{aligned}
I((2,0),\pi_1) &= \frac{\mathbb{E}[b((2,0),\pi_1)]}{1-\gamma((2,0),\pi_1)} \\
&= \frac{p(0)(b(2)+cp(2)b(0)e^{-\beta})}{1-[(1-cp(0))e^{-\beta}+cp(\Delta)e^{-2\beta}]} \\
&= \frac{p(0)(b(2)+cp(2)b(0)e^{-\beta})}{1-e^{-\beta}+cp(0)e^{-\beta}-cp(0)e^{-2\beta}} \\
&= \frac{p(0)(b(2)+cp(2)b(0)e^{-\beta})}{(1-e^{-\beta})(1+cp(0)e^{-\beta})}
\end{aligned}$$

Then we have

$$\begin{aligned}
I((1,0),\pi_1) - I((2,0),\pi_1) &= \frac{p(0)(b(1)+cp(1)b(0)e^{-\beta})}{(1-e^{-\beta})(1+cp(0)e^{-\beta})} - \frac{p(0)(b(2)+cp(2)b(0)e^{-\beta})}{(1-e^{-\beta})(1+cp(0)e^{-\beta})} && [24] \\
&= \frac{p(0)}{(1-e^{-\beta})(1+cp(0)e^{-\beta})} \cdot \big((b(1)+cp(1)b(0)e^{-\beta}) - (b(2)+cp(2)b(0)e^{-\beta})\big) \\
&= \frac{p_T}{(1-e^{-\beta})(1+cp_Te^{-\beta})} \cdot \big(e^{-\beta}+cp_Te^{-\alpha-\beta}-e^{-2\beta}-cp_Te^{-2\alpha-\beta}\big) \\
&= \frac{p_Te^{-\beta}}{(1-e^{-\beta})(1+cp_Te^{-\beta})} \cdot \big(1+cp_Te^{-\alpha}-e^{-\beta}-cp_Te^{-2\alpha}\big) \\
&= \frac{p_Te^{-\beta}}{(1-e^{-\beta})(1+cp_Te^{-\beta})} \cdot \big(1-e^{-\beta}+cp_Te^{-\alpha}(1-e^{-\alpha})\big) \\
&> 0 && [25]
\end{aligned}$$

The final inequality comes from the fact that $\alpha, \beta > 0$. Thus $I((1,0),\pi_1) > I((2,0),\pi_1)$, so $\pi_2 = (1,0)$, and therefore $(1,0) \prec (2,0)$. ◻

The next lemma follows a similar proof structure, this time for $\Delta = 1$.

Lemma 10.4. *Fix $T \in \mathbb{N}$, $p_T \in (0,1]$, $c \in (0,1]$, $\alpha > 0$, and $\beta > \ln(1+cp_Te^{-\alpha})$. Let $D = \mathrm{Ber}(c)$. Then $(1,1) \preceq (2,1)$.*

Proof. Let $S_\pi = \{(0,1),(1,1),(0,2),(2,1)\}$. Then

$$\pi_0 = \operatorname*{arg\,max}_{(h,\Delta)\in S_\pi} I((h,\Delta),\emptyset) = \operatorname*{arg\,max}_{(h,\Delta)\in S_\pi} \frac{p_T e^{-\alpha\Delta-\beta h}}{1-e^{-\beta}} = (0,1)$$

DRAFT

To compute π_1, we compare $I((1,1),\pi_0)$, $I((2,1),\pi_0)$, and $I((0,2),\pi_0)$. The type $(2,1)$ has no potential children in the prefix, so

$$\begin{aligned} I((2,1),\pi_0) &= I((2,1),\emptyset) \\ &= \frac{p(1)b(2)}{1-e^{-\beta}} \\ &= \frac{p_T e^{-\alpha-2\beta}}{1-e^{-\beta}} \end{aligned}$$

The type $(1,1)$ has potential child $(0,1)$ in the prefix. By eq. (23) in lemma 10.1,

$$\begin{aligned} I((1,1),\pi_0) &= \frac{p(1)(b(1)+cp(1)b(0)e^{-\beta})}{(1-e^{-\beta})(1+cp(1)e^{-\beta})} \\ &= \frac{p_T e^{-\alpha}(e^{-\beta}+cp_T e^{-\alpha-\beta})}{(1-e^{-\beta})(1+cp_T e^{-\alpha-\beta})} \end{aligned}$$

Comparing $I((1,1),\pi_0)$ and $I((2,1),\pi_0)$, we have

$$\begin{aligned} I((1,1),\pi_0) - I((2,1),\pi_0) &= \frac{p_T e^{-\alpha}(e^{-\beta}+cp_T e^{-\alpha-\beta})}{(1-e^{-\beta})(1+cp_T e^{-\alpha-\beta})} - \frac{p_T e^{-\alpha-2\beta}}{1-e^{-\beta}} \\ &= \frac{p_T e^{-\alpha-\beta}}{1-e^{-\beta}} \cdot \left(\frac{1+cp_T e^{-\alpha}}{1+cp_T e^{-\alpha-\beta}} - e^{-\beta} \right) \\ &\geq \frac{p_T e^{-\alpha-\beta}}{1-e^{-\beta}} \cdot \left(\frac{1+cp_T e^{-\alpha}}{1+cp_T e^{-\alpha-\beta}} - \frac{1}{1+cp_T e^{-\alpha}} \right) \\ &> 0 \end{aligned}$$

The first inequality is due to the lower bound on β. Thus $I((1,1),\pi_0) > I((2,1),\pi_0)$, so $\pi_1 \neq (2,1)$, leaving $(1,1)$ and $(0,2)$ as the only remaining candidates for π_1. If $\pi_1 = (1,1)$, then $(1,1) \prec (2,1)$, and we are done. Consider the case that $\pi_1 = (0,2)$, and examine π_2. The two remaining candidates are $(1,1)$ and $(2,1)$. Since $\pi_0 = (0,1)$ and $\pi_1 = (0,2)$, the set up is identical to the conclusion of lemma 10.3, substituting $\Delta = 1$ for $\Delta = 0$. Applying this substitution to eq. (24),

$$\begin{aligned} I((1,1),\pi_1) - I((2,1),\pi_1) &= \frac{p(1)(b(1)+cp(1)b(0)e^{-\beta})}{(1-e^{-\beta})(1+cp(1)e^{-\beta})} - \frac{p(1)(b(2)+cp(2)b(0)e^{-\beta})}{(1-e^{-\beta})(1+cp(1)e^{-\beta})} \\ &= \frac{p(1)}{(1-e^{-\beta})(1+cp(1)e^{-\beta})} \cdot \Big((b(1)+cp(1)b(0)e^{-\beta}) - (b(2)+cp(2)b(0)e^{-\beta}) \Big) \\ &= \frac{p_T e^{-\alpha}}{(1-e^{-\beta})(1+cp_T e^{-\alpha-\beta})} \cdot \left(e^{-\beta} + cp_T e^{-\alpha-\beta} - e^{-2\beta} - cp_T e^{-2\alpha-\beta} \right) \\ &= \frac{p_T e^{-\alpha-\beta}}{(1-e^{-\beta})(1+cp_T e^{-\alpha-\beta})} \cdot \left(1 + cp_T e^{-\alpha} - e^{-\beta} - cp_T e^{-2\alpha} \right) \\ &= \frac{p_T e^{-\alpha-\beta}}{(1-e^{-\beta})(1+cp_T e^{-\alpha-\beta})} \cdot \left(1 - e^{-\beta} + cp_T e^{-\alpha}(1-e^{-\alpha}) \right) \\ &> 0 \end{aligned}$$

The final inequality comes from the fact that $\alpha, \beta > 0$. Thus $I((1,1),\pi_1) > I((2,1),\pi_1)$, so $\pi_2 = (1,1)$, which implies that $(1,1) \prec (2,1)$. ☐

In the remaining lemmas, we examine cases where $\Delta > 1$. The following lemma simplifies many of the proofs that follow, by showing that in some regimes, it suffices to compare expected benefits.

Lemma 10.5. *Fix $T \in \mathbb{N}$, $\Delta \in \{0, 1, \ldots, T\}$, $p_T \in (0,1]$, $c \in (0,1]$, and $\alpha, \beta > 0$. Let*

$$S_\pi = \{(1,\Delta), (2,\Delta), (0,1), (1,1), (0,2)\}.$$

Let π be an ordering on S_π that is a subsequence of an optimal priority ordering. Fix a prefix $\pi_0, \pi_1, \ldots, \pi_k$. If the type $(2,\Delta)$ has at least one potential child in the prefix, then $\gamma((2,\Delta),\pi_k) \leq \gamma((1,\Delta),\pi_k)$.

Proof. By definition, for any type (h,Δ) and a prefix $\pi_0, \pi_1, \ldots, \pi_k$,

$$\begin{aligned} \gamma((h,\Delta),\pi_k) &= \sum_{j=1}^{\infty} \Pr[\tau((h,\Delta),\pi_k) = j] e^{-j\beta} \\ &\leq \Pr[\tau((h,\Delta),\pi_k) = 1] e^{-\beta} + \Pr[\tau((h,\Delta),\pi_k) > 1] e^{-2\beta} \end{aligned} \quad [26]$$

The duration $\tau((h,\Delta),\pi_k)$ is strictly greater than 1 if and only if the node of type (h,Δ) is infected and has at least one realized child in the prefix. By definition, a node of type (h,Δ) is infected with probability $p(\Delta)$. Since each potential child is realized with probability c, if the type (h,Δ) has l potential children in the prefix, a node of type (h,Δ) has at least one realized child in the prefix with probability $1-(1-c)^l$. Therefore

$$\Pr[\tau((h,\Delta),\pi_k) > 1] = p(\Delta)(1-(1-c)^l) \quad [27]$$

The type $(1,\Delta)$ has only one potential descendant, the child $(0,1)$, so a $((1,\Delta),\pi_k)$-period has duration at most 2.

$$\begin{aligned} \gamma((1,\Delta),\pi_k) &= \Pr[\tau((1,\Delta),\pi_k) = 1] e^{-\beta} + \Pr[\tau((1,\Delta),\pi_k) = 2] e^{-2\beta} \\ &= \Pr[\tau((1,\Delta),\pi_k) = 1] e^{-\beta} + \Pr[\tau((1,\Delta),\pi_k) > 1] e^{-2\beta} \end{aligned} \quad [28]$$

DRAFT

Then by eq. (27),

$$\begin{aligned}\Pr[\tau((1,\Delta),\pi_k) > 1] &= p(\Delta)(1-(1-c)) \\ &= p(\Delta)c \end{aligned} \quad [29]$$

Consider the type $(2, \Delta)$. By eq. (26),

$$\gamma((2,\Delta),\pi_k) \leq \Pr[\tau((2,\Delta),\pi_k) = 1]e^{-\beta} + \Pr[\tau((2,\Delta),\pi_k) > 1]e^{-2\beta} \quad [30]$$

Since the type $(2, \Delta)$ has at least one potential child in the prefix, by eq. (27) and the fact that $c \in (0, 1]$,

$$\begin{aligned}\Pr[\tau((2,\Delta),\pi_k) > 1] &= p(\Delta)(1-(1-c)^l) \\ &\geq p(\Delta)(1-(1-c)) \\ &= p(\Delta)c \end{aligned} \quad [31]$$

By eq. (29) and eq. (31), $\Pr[\tau((2,\Delta),\pi_k) > 1] \geq \Pr[\tau((1,\Delta),\pi_k) > 1]$. This implies that $\Pr[\tau((2,\Delta),\pi_k) = 1] \leq \Pr[\tau((1,\Delta),\pi_k) = 1]$. Then by eq. (28), eq. (30), and the fact that $e^{-2\beta} < e^{-\beta}$ for $\beta > 0$,

$$\begin{aligned}\gamma((2,\Delta),\pi_k) &\leq \Pr[\tau((2,\Delta),\pi_k) = 1]e^{-\beta} + \Pr[\tau((2,\Delta),\pi_k) > 1]e^{-2\beta} \\ &\leq \Pr[\tau((1,\Delta),\pi_k) = 1]e^{-\beta} + \Pr[\tau((1,\Delta),\pi_k) > 1]e^{-2\beta} \\ &= \gamma((1,\Delta),\pi_k) \end{aligned}$$

Thus $\gamma((2,\Delta),\pi_k) \leq \gamma((1,\Delta),\pi_k)$. □

Lemmas 10.6–10.8 analyze different prefixes of an optimal priority ordering, as referenced by lemma 10.2.

Lemma 10.6. *Fix $T \in \mathbb{N}$, $\Delta \in \{2,\dots,T\}$, $p_T \in (0,1]$, $c \in (0,1]$, $\alpha > 0$, and $\beta > \ln\left(2(1+cp_Te^{-\alpha})/(1-e^{-\alpha})\right)$. Let $D = \mathrm{Ber}(c)$. Let*

$$S_\pi = \{(1,\Delta),(2,\Delta),(0,1),(1,1),(0,2)\}.$$

Let π be an ordering on S_π that is a subsequence of an optimal priority ordering. Suppose $\pi_0 = (0,1)$, $\pi_1 = (0,2)$, and $\pi_2 = (1,1)$. Then $\pi_3 = (1,\Delta)$.

Proof. To determine π_3, we compute the index function for the only remaining types, $(1,\Delta)$ and $(2,\Delta)$. Note that all the potential descendants of types $(1,\Delta)$ and $(2,\Delta)$ are in the prefix. By lemma 10.5, since the type $(2,\Delta)$ has at least one potential child in the prefix, $\gamma((2,\Delta),\pi_2) \leq \gamma((1,\Delta),\pi_2)$. Therefore

$$\begin{aligned} I((1,\Delta),\pi_2) - I((2,\Delta),\pi_2) &= \frac{\mathbb{E}[b((1,\Delta),\pi_2)]}{1-\gamma((1,\Delta),\pi_2)} - \frac{\mathbb{E}[b((2,\Delta),\pi_2)]}{1-\gamma((2,\Delta),\pi_2)} \\ &\geq \frac{1}{1-\gamma((1,\Delta),\pi_2)} \cdot \left(\mathbb{E}[b((1,\Delta),\pi_2)] - \mathbb{E}[b((2,\Delta),\pi_2)]\right) \end{aligned} \quad [32]$$

To compute $\mathbb{E}[b((1,\Delta),\pi_2)]$, we examine the $((1,\Delta),\pi_2)$-period. The period begins by querying the node of type $(1,\Delta)$ on step $t = 0$. With probability $p(\Delta)$ the node is infected, and with probability c it has child $(0,1)$. So with probability $p(\Delta)c$ the node of type $(0,1)$ is queryied on step $t = 1$. Therefore

$$\mathbb{E}[b((1,\Delta),\pi_2)] = p(\Delta)\left(b(1) + cp(1)b(0)e^{-\beta}\right) \quad [33]$$

We follow a similar process to compute $\mathbb{E}[b((2,\Delta),\pi_2)]$. The $((2,\Delta),\pi_2)$-period begins by querying the node of type $(2,\Delta)$ on step $t = 0$, which is infected with probability $p(\Delta)$. Following the priority ordering defined by the prefix, if all potential descendants are realized, then the child of type $(0,2)$ is queried on step $t = 1$, followed by the child of type $(1,1)$ on step $t = 2$; if the child of type $(1,1)$ is infected, then its child of type $(0,1)$ is queried on step $t = 3$. Accounting for the fact that each potential descendant is realized independently with probability c,

$$\begin{aligned} \mathbb{E}[b((2,\Delta),\pi_2)] = p(\Delta)\Big[&b(2) + c\left(p(2)b(0)e^{-\beta} + cp(1)(b(1)e^{-2\beta} + cp(1)b(0)e^{-3\beta}\right) + \\ &(1-c)cp(1)\left(b(1)e^{-\beta} + cp(1)b(0)e^{-2\beta}\right)\Big] \end{aligned} \quad [34]$$

Combining eq. (33) and eq. (34),

$$\begin{aligned} \mathbb{E}[b((1,\Delta),\pi_2)] - \mathbb{E}[b((2,\Delta),\pi_2)] &= p(\Delta)\left(b(1) + cp(1)b(0)e^{-\beta}\right) - \\ &\quad p(\Delta)\Big[b(2) + c\left(p(2)b(0)e^{-\beta} + cp(1)(b(1)e^{-2\beta} + cp(1)b(0)e^{-3\beta})\right) + \\ &\quad (1-c)cp(1)\left(b(1)e^{-\beta} + cp(1)b(0)e^{-2\beta}\right)\Big] \\ &= p(\Delta)\Big[b(1) + cp(1)b(0)e^{-\beta} - \\ &\quad b(2) - cp(2)b(0)e^{-\beta} - c^2p(1)b(1)e^{-2\beta} - c^3p(1)^2b(0)e^{-3\beta} - \\ &\quad (1-c)cp(1)b(1)e^{-\beta} - (1-c)c^2p(1)^2b(0)e^{-2\beta}\Big] \\ &= p_Te^{-\alpha\Delta}\Big[e^{-\beta} + cp_Te^{-\alpha-\beta} - \\ &\quad e^{-2\beta} - cp_Te^{-2\alpha-\beta} - c^2p_Te^{-\alpha-3\beta} - c^3p_T^2e^{-2\alpha-3\beta} - \\ &\quad (1-c)cp_Te^{-\alpha-2\beta} - (1-c)c^2p_T^2e^{-2\alpha-2\beta}\Big] \\ &\geq p_Te^{-\alpha\Delta}\left(cp_Te^{-\alpha-\beta} - cp_Te^{-2\alpha-\beta} - 2cp_Te^{-\alpha-2\beta} - 2c^2p_T^2e^{-2\alpha-2\beta}\right) \end{aligned}$$

DRAFT

The above expression is strictly positive iff

$$cp_T e^{-\alpha-\beta} > cp_T e^{-2\alpha-\beta} + 2cp_T e^{-\alpha-2\beta} + 2c^2 p_T^2 e^{-2\alpha-2\beta} \quad [35]$$

$$1 > e^{-\alpha} + 2e^{-\beta} + 2cp_T e^{-\alpha-\beta} \quad [36]$$

$$1 > e^{-\alpha} + 2e^{-\beta}(1 + cp_T e^{-\alpha}) \quad [37]$$

$$1 - e^{-\alpha} > 2e^{-\beta}(1 + cp_T e^{-\alpha}) \quad [38]$$

$$e^{\beta} > \frac{2(1 + cp_T e^{-\alpha})}{1 - e^{-\alpha}} \quad [39]$$

$$\beta > \ln\left(\frac{2(1 + cp_T e^{-\alpha})}{1 - e^{-\alpha}}\right) \quad [40]$$

Thus the inequality holds due to the bound on β, so $\mathbb{E}[r((1,\Delta),\pi_2)] - \mathbb{E}[r((2,\Delta),\pi_2)] > 0$. Therefore by eq. (32), $I((1,\Delta),\pi_2) > I((2,\Delta),\pi_2)$, so $\pi_3 = (1,\Delta)$. $\square$

Lemma 10.7. *Fix $T \in \mathbb{N}$, $\Delta \in \{2,\dots,T\}$, $p_T \in (0,1]$, $c \in (0,1]$, $\alpha > 0$, and $\beta > \ln\left(2(1 + cp_T e^{-\alpha})/(1 - e^{-\alpha})\right)$. Let $D = \mathrm{Ber}(c)$. Let*

$$S_\pi = \{(1,\Delta), (2,\Delta), (0,1), (1,1), (0,2)\}.$$

Let π be an ordering on S_π that is a subsequence of an optimal priority ordering. Suppose $\pi_0 = (0,1)$ and $\pi_1 = (1,1)$. Then $I((1,\Delta),\pi_1) > I((2,\Delta),\pi_1)$.

Proof. Since the type $(2,\Delta)$ has at least one potential child in the prefix, $\gamma((2,\Delta),\pi_1) \le \gamma((1,\Delta),\pi_1)$. Therefore

$$\begin{aligned} I((1,\Delta),\pi_1) - I((2,\Delta),\pi_1) &= \frac{\mathbb{E}[b((1,\Delta),\pi_1)]}{1 - \gamma((1,\Delta),\pi_1)} - \frac{\mathbb{E}[b((2,\Delta),\pi_1)]}{1 - \gamma((2,\Delta),\pi_1)} \\ &\ge \frac{1}{1 - \gamma((1,\Delta),\pi_1)} \cdot \left(\mathbb{E}[b((1,\Delta),\pi_1)] - \mathbb{E}[b((2,\Delta),\pi_1)]\right) \quad [41] \end{aligned}$$

The type $(1,\Delta)$ has one potential child $(0,1)$ in the prefix and no other descendants, so

$$\mathbb{E}[b((1,\Delta),\pi_1)] = p(\Delta)[b(1) + cp(1)b(0)e^{-\beta}] \quad [42]$$

To compute $\mathbb{E}[b((2,\Delta),\pi_2)]$ we examine the $((2,\Delta),\pi_2)$-period. The period begins by querying the node of type $(2,\Delta)$ on step $t = 0$. With probability $p(\Delta)$ the node is infected, and with probability c it has child $(1,1)$, which is then queried on step $t = 1$. With probability $p(\Delta)$ the node $(1,1)$ is infected, and with probability c it has child $(0,1)$, which is then queried on step $t = 2$. Thus

$$\mathbb{E}[b((2,\Delta),\pi_1)] = p(\Delta)[b(2) + cp(1)\left(b(1)e^{-\beta} + cp(1)b(0)e^{-2\beta}\right)] \quad [43]$$

Combining eq. (42) and eq. (43),

$$\begin{aligned} \mathbb{E}[b((1,\Delta),\pi_1)] - \mathbb{E}[b((2,\Delta),\pi_1)] &= p(\Delta)[b(1) + cp(1)b(0)e^{-\beta} - b(2) - cp(1)\left(b(1)e^{-\beta} + cp(1)b(0)e^{-2\beta}\right)] \\ &= p_T e^{-\alpha\Delta}[e^{-\beta} + cp_T e^{-\alpha-\beta} - e^{-2\beta} - cp_T e^{-\alpha-2\beta} - c^2 p_T^2 e^{-2\alpha-2\beta}] \\ &\ge p_T e^{-\alpha\Delta}[cp_T e^{-\alpha-\beta} - cp_T e^{-\alpha-2\beta} - c^2 p_T^2 e^{-2\alpha-2\beta}] \end{aligned}$$

The above expression is strictly positive iff

$$\begin{aligned} cp_T e^{-\alpha-\beta} &> cp_T e^{-\alpha-2\beta} + c^2 p_T^2 e^{-2\alpha-2\beta} \\ 1 &> e^{-\beta} + cp_T e^{-\alpha-\beta} \\ 1 &> e^{-\beta}(1 + cp_T e^{-\alpha}) \\ e^{\beta} &> 1 + cp_T e^{-\alpha} \\ \beta &> \ln(1 + cp_T e^{-\alpha}) \end{aligned}$$

The inequality holds due to the bound on β, so $\mathbb{E}[b((1,\Delta),\pi_1)] - \mathbb{E}[b((2,\Delta),\pi_1)] > 0$. Therefore by eq. (41), $I((1,\Delta),\pi_1) > I((2,\Delta),\pi_1)$. $\square$

The following lemma is nearly identical to lemma 10.6 and references the prior lemma for many computations. The key difference between the two settings is that in the prefix that lemma 10.8 considers, $(1,1) \prec (0,2)$, whereas the opposite holds in lemma 10.6. This difference in prefix changes the computation of the index function for $(2,\Delta)$.

Lemma 10.8. *Fix $T \in \mathbb{N}$, $\Delta \in \{2,\dots,T\}$, $p_T \in (0,1]$, $c \in (0,1]$, $\alpha > 0$, and $\beta > \ln\left(2(1 + cp_T e^{-\alpha})/(1 - e^{-\alpha})\right)$. Let*

$$S_\pi = \{(1,\Delta), (2,\Delta), (0,1), (1,1), (0,2)\}.$$

Let π be an ordering on S_π that is a subsequence of an optimal priority ordering. Suppose $\pi_0 = (0,1)$, $\pi_1 = (1,1)$, and $\pi_2 = (0,2)$. Then $\pi_3 = (1,\Delta)$.

Proof. To select π_3, we compute the index function for the two remaining types, $(1,\Delta)$ and $(2,\Delta)$. Following the same argument as in lemma 10.6, this reduces to comparing expected benefits over an $((h,\Delta),\pi_2)$-period. Therefore

$$\begin{aligned} I((1,\Delta),\pi_2) - I((2,\Delta),\pi_2) &= \frac{\mathbb{E}[b((1,\Delta),\pi_2)]}{1 - \gamma((1,\Delta),\pi_2)} - \frac{\mathbb{E}[b((2,\Delta),\pi_2)]}{1 - \gamma((2,\Delta),\pi_2)} \\ &\ge \frac{1}{1 - \gamma((1,\Delta),\pi_2)} \cdot \left(\mathbb{E}[b((1,\Delta),\pi_2)] - \mathbb{E}[b((2,\Delta),\pi_2)]\right) \quad [44] \end{aligned}$$

DRAFT

Just as in lemma 10.6, the prefix includes $(0,1)$, so the computation of $\mathbb{E}[b((1,\Delta),\pi_2)]$ is also the same.

$$\mathbb{E}[b((1,\Delta),\pi_2)] = p(\Delta)\left(b(1) + cp(1)r(0)e^{-\beta}\right) \tag{45}$$

To compute $\mathbb{E}[b((2,\Delta),\pi_2)]$ we examine the $((2,\Delta),\pi_2)$-period. The period begins by querying the node of type $(2,\Delta)$ on step $t=0$, which is infected with probability $p(\Delta)$. Following the priority ordering defined by the prefix, if all potential descendants are realized, then the child of type $(1,1)$ is queried on step $t=1$; if it is infected, then the node of type $(0,1)$ is queried on step $t=2$, followed by the child of type $(0,2)$ on step $t=3$. If the child of type $(1,1)$ is not infected, then the node of type $(0,1)$ is skipped, and the child of type $(0,2)$ is queried on step $t=2$. Accounting for the fact that each potential descendant is realized independently with probability c,

$$\begin{aligned}\mathbb{E}[b((2,\Delta),\pi_2)] = p(\Delta)\Big[b(2) + c\Big[p(1)\left(b(1)e^{-\beta} + cp(1)b(0)e^{-2\beta} + cp(2)b(0)e^{-3\beta}\right) + \\ (1-p(1))cp(2)b(0)e^{-2\beta}\Big] + (1-c)cp(2)b(0)e^{-\beta}\Big]\end{aligned} \tag{46}$$

Combining eq. (45) and eq. (46),

$$\begin{aligned}\mathbb{E}[b((1,\Delta),\pi_2)] - \mathbb{E}[b((2,\Delta),\pi_2)] &= p(\Delta)\left(b(1) + cp(1)b(0)e^{-\beta}\right) - \\ &\quad p(\Delta)\Big[b(2) + c\Big[p(1)\left(b(1)e^{-\beta} + cp(1)b(0)e^{-2\beta} + cp(2)b(0)e^{-3\beta}\right) + \\ &\quad (1-p(1))cp(2)b(0)e^{-2\beta}\Big] + (1-c)cp(2)b(0)e^{-\beta}\Big] \\ &= p_T e^{-\alpha\Delta}\Big[e^{-\beta} + cp_T e^{-\alpha-\beta} - \\ &\quad e^{-2\beta} - cp_T e^{-\alpha-2\beta} - c^2p_T^2e^{-2\alpha-2\beta} - c^2p_T^2e^{-3\alpha-3\beta} - \\ &\quad (1-p_Te^{-\alpha})c^2p_Te^{-2\alpha-2\beta} - (1-c)cp_Te^{-2\alpha-\beta}\Big] \\ &\geq p_Te^{-\alpha\Delta}\Big[cp_Te^{-\alpha-\beta} - (1-c)cp_Te^{-2\alpha-\beta} - \\ &\quad \left(cp_Te^{-\alpha-2\beta} + (1-p_Te^{-\alpha})c^2p_Te^{-2\alpha-2\beta}\right) - \\ &\quad \left(c^2p_T^2e^{-2\alpha-2\beta} + c^2p_T^2e^{-3\alpha-3\beta}\right)\Big] \\ &\geq p_Te^{-\alpha\Delta}\Big[cp_Te^{-\alpha-\beta} - cp_Te^{-2\alpha-\beta} - 2cp_Te^{-\alpha-2\beta} - 2c^2p_T^2e^{-2\alpha-2\beta}\Big]\end{aligned}$$

By eqs. (35)–(40), the above expression is strictly positive, so $\mathbb{E}[b((1,\Delta),\pi_2)] - \mathbb{E}[b((2,\Delta),\pi_2)] > 0$. Therefore by eq. (44) $I((1,\Delta),\pi_2) > I((2,\Delta),\pi_2)$, so $\pi_3 = (1,\Delta)$. □

DRAFT
